# Characteristics of patients with low back and leg pain seeking treatment in primary care: baseline results from the ATLAS cohort study

**DOI:** 10.1186/s12891-015-0787-8

**Published:** 2015-11-04

**Authors:** Kika Konstantinou, Kate M. Dunn, Reuben Ogollah, Steven Vogel, Elaine M. Hay, On the behalf of the ATLAS study research team

**Affiliations:** Arthritis Research UK Primary Care Centre, Research Institute for Primary Care & Health Sciences, Keele University, Staffordshire, ST5 5BG UK; British School of Osteopathy, London, UK

**Keywords:** Epidemiology, Back pain, Leg pain, Sciatica, Cross-sectional

## Abstract

**Background:**

Patients with back pain radiating to the leg(s) report worse symptoms and poorer recovery than those with back pain alone. Robust evidence regarding their epidemiological profile is lacking from primary care, the setting where most of these patients will present and be managed. Our objective was to describe the characteristics of patients with back and leg pain, including sciatica, seeking treatment in primary care.

**Methods:**

Adults visiting their general practitioner with back and leg pain, of any duration and severity, were invited to participate. Participants completed questionnaires, underwent clinical assessments and received MRI scans. Characteristics of the sample are described, and differences between patients diagnosed with referred leg pain and those with sciatica are analysed.

**Results:**

Six hundred nine patients participated; 62.6 % were female, mean (SD) age 50.2 (13.9). 67.5 % reported pain below the knee, 60.7 % were in paid employment with 39.7 % reporting time off work. Mean disability (RMDQ) was 12.7 (5.7) and mean pain intensity was 5.6 (2.2) and 5.2 (2.4) for back and leg respectively. Mean sciatica bothersomeness index (SBI) was 14.9 (5.1). Three quarters (74.2 %) were clinically diagnosed as having sciatica. In the sciatica group, leg pain intensity, neuropathic pain, pain below the knee, leg pain worse than back pain, SBI and positive MRI findings were significantly higher as compared to patients with referred leg pain.

**Conclusions:**

This primary care cohort reported high levels of disability and pain. This is the first epidemiological study of unselected primary care patients seeking healthcare for back and leg pain. Follow-up of this cohort will investigate the prognostic value of their baseline characteristics. This new information will contribute to our understanding of the characteristics and clinical features of this population, and will underpin future research aimed at defining prognostic subgroups to enable better targeting of health care provision.

## Background

In primary care, approximately 60 % of patients presenting with low back pain (LBP) also report pain in the leg(s) [1]. Leg pain associated with LBP is generally considered to be either referred or radicular pain. The latter is commonly labelled sciatica and is often characterised by pain radiating to below the knee, into the foot and toes, and may be accompanied by objective findings of nerve root entrapment such as sensory deficits, reflex changes or muscle weakness [2]. The most common reasons for sciatica are a disc bulge/prolapse or stenosis (either of the central canal or the foramen) impinging or irritating a nerve root(s). Referred leg pain from the low back is unrelated to nerve root involvement and is considered as pain referred from any other structure such as muscle, ligament, joint or intervertebral disc. It is generally acknowledged that the differentiation between sciatica and referred leg pain is not always straightforward in clinical practice, but ultimately it is a clinical diagnosis [3, 4]. Overall, the literature indicates that patients who complain of back and leg pain and/or sciatica suffer more severe pain and disability, take longer to recover and incur most of the indirect costs and lost workdays compared to those with back pain alone [1, 5–7]. Hence most back pain national and international guidelines recommend assessing, diagnosing and addressing back related leg pain early in the presentation of patients complaining of back problems, so that treatment can be prioritised and delivered to this subset of LBP patients with increased risk of poor prognosis [8]. However, there is an inherent contradiction in the guidelines as despite the call for accurate and early diagnosis of low back related leg pain, the recommendations for initial treatment of patients with leg pain, including sciatica, appear to be similar to those for non-specific LBP.

One reason for this confusion and lack of clarity in the guidelines relates to the poor evidence base regarding outcome and treatment effectiveness for LBP patients with related leg pain.

Few studies exist which describe the characteristics and clinical course of the full range of patients seeking care for low back and leg pain/sciatica. Most studies focus on patients with back pain alone, include mixed populations with back and leg pain (without differentiating between them), or are concerned with describing the characteristics of highly selected populations from tertiary care settings (including surgical candidates). Robust evidence regarding the epidemiology of low back related leg pain (including sciatica) is lacking from primary care, the setting where the majority of these patients will present and be managed.

Information about the broad group of patients with LBP and leg pain presenting to primary care will contribute to our understanding of the characteristics and clinical features of this population, and will help patients, health care practitioners and managers better define and deliver appropriate health care provision.

The main objective of this study is to describe the characteristics, imaging findings and clinician defined specific diagnosis, in patients with low back and leg pain seeking treatment in a primary care setting.

We also investigated key differences in patients’ characteristics between the groups with and without a clinical diagnosis of sciatica as clinicians consider them to have different natural and clinical courses, and potential management options for each group also differ. For consistency we use the terms “sciatica” and “referred leg pain” throughout this paper to denote those with, and without, clinically diagnosed presence or absence of spinal nerve root involvement.

## Methods

### Study design and participants

This paper presents baseline data from a prospective observational cohort of primary care consulters with back and leg pain/sciatica (ATLAS). The protocol for the ATLAS study (**A**ssessment and **T**reatment of **L**eg pain **A**ssociated with the **S**pine) has been published [9]. A brief overview of the methods is provided here. Ethical Approval for this study was obtained by the South Birmingham Research Ethics Committee (REC ref. 10/H1207/82).

Participants were recruited between April 2011 and March 2013 from 17 primary care practices in North Staffordshire and Stoke-on-Trent, UK. The study population consisted of adults aged 18 years and over, visiting their General Practitioner (GP) with LBP and associated leg pain of any duration and intensity but without spinal ‘red flags’ or serious physical or mental comorbidities thought likely to preclude their ability to attend a research clinic (see Appendix for exclusion criteria).

**Fig. 1:**
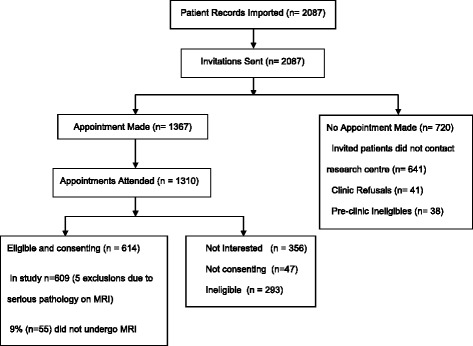
ATLAS study flow diagram

Leg pain was defined as pain spreading from the back beyond the gluteal fold to anywhere in the leg. In this context “pain” was taken to include all unpleasant/abnormal sensations such as ‘pins and needles’ or numbness. Potentially eligible patients were identified at the GP consultation by the use of Read codes [10] indicating a low back with leg pain consultation. This information was downloaded weekly and patients were sent a letter from their primary care practice, along with information about the ATLAS research study, inviting them to telephone the research clinic administrator to find out more about the study and make an appointment at the clinic (for full details of the recruitment and consent procedures see the study protocol [9]).

All patients attending the research clinic and after giving written consent, received a standardised assessment by a study physiotherapist to confirm eligibility and agree a treatment plan. All study physiotherapists (*n* = 7) performing the assessments were senior staff with experience in the assessment and treatment of spinal musculoskeletal conditions^1^ and had been given training in the procedures of the study. Diagnosis of sciatica or referred leg pain was based on the clinical opinion of the assessing clinician, no specific criteria were imposed on the assessors for reaching their diagnostic opinion. Consenting, eligible participants received an MRI scan within 2 weeks of their baseline assessment, providing there were no clinical contra-indications to the procedure (for full details of the MRI protocol see the study protocol [9]). The MRI findings did not influence initial clinical diagnosis.

Participation in the study did not confer any specific treatment advantage for the patients, which followed care pathways based on current best clinical evidence, practice guidelines and local services and resources.

### Measurements

Self-reported measures were collected with postal questionnaires at baseline, and 4 and 12 months later. Clinical assessment findings were collected at baseline. (For full details of study measures see protocol [9]). Care pathways were recorded at end of treatment.

### Sociodemographic variables

Information was collected on age, gender, employment status, type of work, interference of pain with work performance [11, 12] (measured on a 0 to 10 scale where 0 is ‘not at all’ and 10 is ‘the pain is so bad that I am unable to do my job’), time off work (days), smoking and Body Mass Index (BMI) (height and weight measured in clinic).

### Low back and leg pain/sciatica characteristics

Disability was measured with the Roland Morris Disability Questionnaire (RMDQ) leg pain version which has 23 items scored from 0 to 23 with higher scores indicating higher disability [13, 14]. Back pain intensity was measured using the mean of three 0 to 10 numerical rating scales for least, current and usual back pain over the previous 2 weeks [15]. Leg pain intensity was measured using the mean of three 0 to 10 numerical rating scales for least, usual and current leg pain over the previous 2 weeks. Neuropathic pain was measured using the self-report Leeds Assessment Neuropathic Symptoms and Signs (S-LANSS) which has seven items and a possible range from 0 to 24, with a score of 12 or more indicating possible neuropathic pain [16]. Sciatic symptoms were assessed with the Sciatica Bothersomeness Index (SBI), a self-scored questionnaire assessing four symptoms; leg pain, numbness or tingling in the leg, foot, or groin, weakness in the leg or foot and back or leg pain while sitting. The SBI is a composite score of these four symptoms; each one scored on a 0–6 scale with possible total score of 24 with higher scores indicating worse symptoms [14]. Data were collected on symptom duration, whether this was a first ever episode of back and leg pain, whether pain extended below the knee and whether leg pain was worse than back pain. The STarT Back screening tool for risk of persistent LBP disability was also included [17].

### Psychological and other health related variables

Anxiety and depression were measured using the Hospital Anxiety and Depression scale (HADs) with scores from 0 to 21, higher scores indicate higher levels of anxiety/depressive symptoms, with a cut-off point of ≥11 considered to indicate ‘probable depression/anxiety’ [18]. Pain self-efficacy was measured with the Pain Self-Efficacy Questionnaire (PSEQ) with scores from 0 to 60; higher scores reflect stronger self-efficacy beliefs [19]. Illness perceptions were measured with the Musculoskeletal Illness Perceptions Questionnaire (IPQ-R) Short-Form (adapted from Moss-Morris et al. 2002) [20]. The IPQ-R comprises seven dimensions which assess people’s beliefs about: timeline– acute/chronic (illness duration), consequences (the impact of the illness on their life), personal control (how much influence they have over the illness), treatment control (how amenable the illness is to medical intervention), illness coherence (how well they understand the illness) and timeline–cyclical (whether the illness trajectory is constant or cyclical), as well as emotional representations (the emotional impact of the illness). High scores on a particular dimension represent strong perceptions of it. In addition, the IPQ-R also assesses beliefs about identity (symptom attribution) and causes (of the illness). High scores on the identity dimension represent strongly held beliefs about the number of symptoms attributed to the illness (see footnote)^2^. General health was captured with the SF-1 (excellent to poor) [21] and EQ5D [22]. Co-morbidities were recorded by self-report from a list of five possible conditions (chest problems, heart problems, hypertension, diabetes, circulation problems in legs). Sleep disturbances due to low back and leg pain were also recorded.

### Clinical assessment variables

Participants underwent a standardised clinical assessment including subjective history and physical examination and an MRI scan. The assessors were asked to indicate whether the presence of leg pain was due to sciatica or to referred leg pain and their confidence (percentage) in their clinical impression/diagnosis and to indicate a specific diagnosis for the sciatic symptoms (i.e. disc prolapse, stenosis) (for full details on assessors training see study protocol [9]).

### Scoring of MRI

MRIs were scored by a single assessor: a senior consultant musculoskeletal radiologist (JS) blind to any specific clinical information relating to patient’s symptoms other than the patient presentation with back and leg pain (not stating which leg). The assessor provided a clinical report indicating the presence/absence of definite or possible nerve root compression by lumbar spinal level (3 lower lumbar levels) and side (right/left) and the reason(s) for it if present (e.g. disc prolapse, stenotic features, etc.) as per normal practice in radiological reporting.

### Statistical analysis

Descriptive data for the whole sample are presented as means and standard deviations for continuous variables and as frequency counts and percentages for categorical variables.

Key characteristics were compared between patients with and without a clinical diagnosis of sciatica using two-sample t-tests for continuous variables (with normality and homogeneity of variance assumptions tested) and Pearson’s Chi-squared test for categorical variables (Fisher’s exact test used for cell frequencies <5). The key variables included: age, gender, smoking, BMI, back and leg pain scores, disability, anxiety and depression, work interference and any time off work, first ever episode, worse pain in low back or leg, pain below knee, neuropathic pain score, sciatica bothersomeness index score and sleep disturbance, STarT Back tool score, MRI findings.

## Results

### Study sample

Two thousand and eighty seven potentially eligible patients identified at primary care visit were invited to the study; 1310 attended the research clinic, and 614 consented and were eligible to participate. The detailed flow of patients into the study is presented in Fig. 1. The reasons for ineligibility are presented in Appendix, with the most common reason being absence of leg pain and/or complete recovery from symptoms.

Of the 614 patients in the study, five patients were (subsequently) excluded from the study and referred on to secondary care services following their MRI results, as they were found to have initially unsuspected serious/specific pathologies. These were: avascular necrosis of the femoral head (AVN), thoracic spinal cord compression, widespread metastatic cancer, large benign pelvic cyst/tumour. All four cases presented at baseline assessment as sciatic pain, and one case of inflammatory arthritis presented as referred leg pain. Based on the above numbers the rate of unsuspected specific/serious pathologies causing back and leg pain in this sample of primary care consulters was 0.8 % (5/614). A further three patients were incidentally found to have stage IV renal carcinomas (pain free) and were referred to the appropriate secondary care services but remained in the study as these were not spinal pain related findings. The final sample in the study was 609 patients. Overall, 9.1 % of the participants (55/614) did not have the MRI scan, most of them declined due to claustrophobia, plus four having contraindications to the procedure.

### Patients’ baseline characteristics

The sociodemographic, physical and psychological profiles, including imaging findings, of all study participants are presented in Tables 1, 2 and 3 respectively.

**Table 1:** Baseline socio-demographic characteristics of participants

Socio-demographics (Denominator^a^)	*n* = 609
Age (years) (609), mean (SD)	50.2 (13.9)
Age categories (609)	
18–34	91 (14.9)
35–44	136 (22.3)
45–54	152 (25.0)
55–64	138 (22.7)
65+	92 (15.1)
Gender (609), Female	381 (62.6)
Current smoker (609)	194 (31.9)
BMI categories (kg/m^2^) (607)	
Normal (18.5 to <25)	136 (22.4)
Overweight (25 to <30)	223 (36.7)
Obese (30 to <40)	205 (33.8)
Morbidly obese (40+)	43 (7.1)
Currently in paid job (605)	367 (60.7)
Self-certified time off work or given sick note due to current episode (365)^b^	144 (39.7)
Duration (days) self-certified off work (106)	
1 to 3 days	33 (31.1)
4 to 6 days	29 (27.4)
7 days	44 (41.5)
Duration (days) given sick note (98)	
1 to 14 days	53 (53.1)
15 to 21 days	10 (10.2)
22–28 days	18 (18.4)
More than 28 days	18 (18.4)
National statistics socio-economic classification (based on current or most recent paid job) (593)	
Higher managerial, administrative and professional occupations	129 (21.8)
Intermediate occupations	158 (26.6)
Routine and manual occupations	283 (47.7)
Never worked and long-term unemployed	23 (3.9)
Back/leg pain interference with work performance (361), mean (SD)^b^	5.9 (2.9)
Main employment activity (work load)	
Predominately sitting (>4 h) (448)	143 (31.9)
Predominately standing (>4 h) (451)	233 (51.7)
Manual lifting; <10 kg(>4 h) (446)	60 (13.5)
Manual lifting; >10 kg (>4 h) (445)	42 (9.4)

All figures are frequencies (percentages) unless stated otherwise as mean (SD)

^a^Denominator varies for some participants due to missing data or not applicable case

^b^Applicable to only those currently working

**Table 2:** Baseline physical (self-reported) and MRI characteristics of participants

Physical Characteristics (Denominator^a^)	*n* = 609
Pain and function	
RMDQ disability score (0–23) (609), mean (SD)	12.7 (5.7)
Back pain intensity (609), mean (SD)	5.6 (2.2)
Leg pain intensity (608), mean (SD)	5.2 (2.4)
Pain below knee (584)	394 (67.5)
Leg pain worse than back pain (609)	280 (46.0)
Sleep disturbance due to back/leg pain^b^(609)	428 (70.3)
First ever episode of back/leg pain (609)	58 (9.5)
Duration of symptoms	
Back pain current episode (607)	
Less than 6 weeks	218 (35.9)
6–12 weeks	126 (20.8)
>3 months	263 (43.3)
Leg pain current episode (583)	
Less than 6 weeks	251 (43.1)
6–12 weeks	120 (20.6)
>3 months	212 (36.4)
Start Back prognostic risk score (589)	
Low Risk	82 (13.9)
Medium Risk	276 (46.9)
High Risk	231 (39.2)
Sciatica Bothersomeness Index (SBI), mean (SD)	
Leg pain (583)	4.3 (1.5)
Numbness or tingling in leg, foot or groin (583)	3.2 (2.1)
Weakness in leg or foot (582)	2.6 (2.1)
Back or leg pain while sitting	4.1 (1.6)
*Composite score (582)*	14.2 (5.4)
S-LANSS; neuropathic pain score (>12) (607)	293 (48.1)
Imaging(553)	
Positive MRI findings for nerve root compression	297 (53.7)

All figures are frequencies (percentages) unless stated otherwise as mean (SD)

^a^Denominator varies for some participants due to missing data or not applicable case

^b^Information on back and/or leg pain associated sleep disturbance was collected as part of the clinical assessment (yes/no category)

**Table 3:** Baseline self-reported psychological and health characteristics of participants

Psychological and other health measures (Denominator^a^)	*n* = 609
HADs anxiety subscale (607)	
Normal	316 (52.1)
Mild/possible case	120 (19.8)
Probable/moderate/severe case	171 (28.2)
HADs depression subscale (609)	
Normal	392 (64.4)
Mild/possible case	119 (19.5)
Probable/moderate/severe case	98 (16.1)
Pain self-efficacy score (593), mean (SD)	34.1 (14.6)
Illness perceptions questionnaire-short form (IPQ-R)	
Timeline-‘back/leg pain will last for a long time (agree or strongly agree) (609)	345 (56.7)
Personal control-‘what I do can determine whether back/leg pain gets better (agree or strongly agree) (605)	367 (60.7)
Identity score (0–7) (584), mean (SD)	5.9 (1.3)
General Health (608)	
Excellent/very good	146 (24.0)
Good	241 (39.5)
Fair	172 (28.3)
Poor	50 (8.2)
*Co-morbidities* ^b^ *(609)*	
None	371 (60.9)
One other health problem	158 (25.9)
Two or more other health problems	80 (13.1)
EQ—5D—3L summary index (590)	0.44 (0.32)

All figures are frequencies (percentages) unless stated otherwise as mean (SD)

^a^Denominator varies for some participants due to missing data or not applicable case

^b^The health problems include chest problems, heart problems, raised blood pressure, diabetes, and circulation problems in the leg

The mean age of participants was 50.2 (13.9) years with a range of 18 to 82 years; 15.1 % of participants were 65 years of age or over. 62.6 % were female. Mean BMI was 29.9 (7.0). 60.7 % (367) were in paid employment and 47.7 % of the cohort were in routine and manual occupations. Over a third of the cohort (39.3 %), were not currently in a paid job. Of those in employment, 39.7 % reported having time off work due to their current episode of back and leg pain, either self-certified or with formal sickness certification. Almost a third (31.9 %), were current smokers.

Only 9.5 % of patients reported that this was their first episode of back and/or leg pain and nearly half, had symptoms for over 3 months. Mean (SD) disability score, as measured by the RMDQ, was 12.7 (5.7). Mean leg pain intensity was 5.2 (2.4) and 67.5 % reported pain below the knee. Mean SBI composite score was 14.2 (5.4). Almost half (48.1 %) scored ≥12 points on the S-LANSS, indicating possible neuropathic pain. Most patients (70.3 %) reported sleep disturbances due to their back and/or leg pain. Mean scores for anxiety and depression as measured by the HADs were 7.8 (4.2) and 6.4 (4.0) respectively. 86.1 % (medium risk; 46.9 %, high risk; 39.2 %) were at risk of future persistent disability as measured by the STarT Back screening tool.

### Clinical impression/diagnosis and sciatica prevalence

Based on the clinical assessment, 74.2 % (452/609) of the sample was diagnosed as having sciatica. Diagnosis was reached with variable degrees of confidence (range 50 to 100 %). In 70 % of all cases the confidence in clinical diagnosis was ≥80 % with a similar distribution of confidence range for patients with and without sciatica. Specific clinical diagnoses for the sciatica group were: disc prolapse; 53.7 %, stenosis (central or foraminal); 10.6 % and in 35.7 % no specific pathoanatomical diagnosis was made for the presenting sciatic symptoms.

### MRI findings

Of the 554 patients having an MRI scan, 37.8 % were found to have evidence of definite nerve root compression and 15.9 % of possible nerve root compression, on the relevant side. Among those with a clinical diagnosis of sciatica, 60.7 % had definite or possible nerve root compression on MRI compared to 32.4 % of those diagnosed with referred leg pain.

In the sciatica group with positive MRI findings (*n* = 252), 79.8 % had a disc prolapse accounting for nerve root compression, including three cases of cauda equina compression. Stenosis was reported in 18.2 %, with another three cases of nerve root compression due to osteophytes, one due to a synovial cyst and one due to epidural lipomatosis constricting the theca.

In the referred leg pain group with positive MRI findings (*n* = 45), 68.9 % had a disc prolapse and 24.4 % had stenosis.

### Diagnostic groups

Table 4 presents data on key variables from self-report, clinical assessment (including neurological examination findings) and imaging findings, between patients clinically diagnosed with or without sciatica.

**Table 4:** Comparison of key characteristics for patients diagnosed with or without sciatica

Characteristics	Sciatica pain	Referred pain	Sig.*
	*n* = 452 (74.2 %)	*n* = 157 (25.8 %)	
Socio-demographics			
Age (years), mean (SD)	50.4 (14.0)	49.4 (13.7)	0.451
Gender, Female	276 (61.1)	105 (66.9)	0.194
BMI	29.9 (6.3)	30.0 (8.7)	0.906
Current smoker	151 (33.4)	43 (27.4)	0.163
Back/leg pain interference with work performance, mean (SD)^a^	6.0 (2.9)	5.4 (3.0)	0.073
Self-certified time off work or given sick note due to current episode (365)^a^	111 (40.8)	33 (36.3)	0.443
Pain and function			
RMDQ disability score (0–23), mean (SD)	12.9 (5.7)	12.0 (5.7)	0.093
Back pain intensity, mean (SD)	5.6 (2.2)	5.4 (2.1)	0.413
Leg pain intensity, mean (SD)	5.6 (2.3)	4.1 (2.3)	<0.001
Pain below knee	333 (76.6)	61 (40.9)	<0.001
Leg pain is worse	252 (55.8)	28 (17.8)	<0.001
Sleep disturbance due to back/leg pain	325 (72)	103 (66)	0.447
EQ—5D summary index	0.4 (0.3)	0.5 (0.3)	0.391
Start Back risk score			0.086
Low	53 (12.1)	29 (19.1)	
Medium	212 (48.5)	64 (42.1)	
High	172 (39.4)	59 (38.8)	
Sciatica Bothersomeness Index, mean (SD)			
Leg pain	4.5 (1.4)	3.6 (1.6)	<0.001
Numbness or tingling in leg, foot or groin	3.5 (2.0)	2.4 (2.1)	<0.001
Weakness in leg or foot	2.8 (2.0)	2.0 (2.1)	<0.001
Back or leg pain while sitting	4.1 (1.6)	4.1 (1.7)	0.084
*Composite score*	14.9 (5.1)	12.2 (5.4)	*<0.001*
S-LANSS; neuropathic pain score (≥12)	232 (51.6)	61 (39.0)	*<0.007*
Psychological measures			
*HADs anxiety subscale*			0.023
Mild/possible case	86 (19.1)	34 (21.8)	
Probable/moderate/severe case	116 (25.7)	55 (35.3)	
*HADs depression subscale*			0.325
Mild/possible case	82 (18.1)	37 (23.4)	
Probable/moderate/severe case	75 (16.6)	23 (14.7)	
MRI findings(553)			<0.001
Nerve root compression	252 (60.7)	45 (32.4)	
Normal	163 (39.3)	94 (67.6)	
Neurological examination findings (609)			
*Muscle weakness* ^b^			<0.001
None	347 (76.8)	157 (100)	
Mild (4)	92 (20.4)	0 (0.0)	
Severe (≤3)	13 (2.9)	0 (0.0)	
*Reflex change*			<0.001
None	341 (75.4)	149 (94.9)	
Slightly reduced	30 (6.6)	0 (0.0)	
Significantly reduced	19 (4.2)	3 (1.9)	
Absent	62 (13.7)	5 (3.2)	
*Sensory change*			<0.001
None	226 (50.0)	130 (82.8)	
Reduced pin/prick	175 (38.7)	26 (16.6)	
Loss to pin/prick	51 (11.3)	1 (0.6)	
*Neural tension tests (any positive test)* ^c^	324 (71.7)	11 (7.0)	<0.001

All figures are frequencies (percentages) unless stated otherwise as mean (SD)

^a^Applicable to those currently in paid job

^b^Muscle strength tested according to the Oxford scale where;

1. Flicker of movement

2. Through full range actively with gravity counterbalanced

3. Through full range actively against gravity

4. Through full range actively against some resistance

5. Through full range actively against strong resistance

^c^Neural tension tests; straight leg raise, femoral stretch, slump test

*Significance *p*-value for the difference between participants diagnosed as having sciatica symptoms and those diagnosed as having referred leg pain based on Chi-squared test for categorical variables and 2-sample *t*-test for continuous variables

Those with sciatica had higher levels of leg pain and more often reported below the knee pain and leg pain worse than back pain. These patients also scored higher on the SBI and the neuropathic pain scales. MRI findings of spinal nerve root compression were more often present for patients diagnosed with sciatica.

### Care pathways

The majority of patients in this cohort received physiotherapy interventions with only 4.4 % receiving a single treatment session. 11.5 % were referred to secondary care services for consideration of more invasive interventions such as spinal injections and surgery. The majority of the secondary care referrals were made for patients in the sciatica group (83.0 %).

### Selection bias

Although it is not possible to provide detailed data on the characteristics of non-responders to the clinic invitation, those who were non-eligible or declined participation, comparison of age, gender and area deprivation for participants and non-participants shows reasonable comparability in these three key baseline characteristics. Slightly more women (63 % vs 57 %), slightly younger patients (mean age 50 vs 55) and slightly more patients from least deprived area tertile (36 % vs. 31 %) participated, which is a common finding in the literature. There were no significant differences in characteristics between patients undergoing an MRI and those that did not (data not presented).

## Discussion

In this paper we describe the characteristics of 609 primary care patients seeking care for symptoms of back and leg pain including sciatica. To our knowledge this is the first, primary care cohort of patients seeking care for back and leg pain, including symptoms of any duration and severity, to be described in detail.

In this group of patients with back and leg pain mean levels of pain and disability were moderate, nearly half had symptoms for over 3 months, over two thirds reported pain related sleep disturbances, and the majority scored at medium or high risk of future back pain related disability. Nearly three quarters of the participants were clinically diagnosed as having sciatica, with diagnostic confidence of 80 % and over for the majority. Approximately half of this cohort was likely to have pain of neuropathic nature as measured with self-reported validated scales. MRI findings of nerve root compression were present in just over half of the participants. Unsuspected serious pathologies (including incidental finding of kidney malignancies), detected because of the MRI scans, were 1.3 %.

Only 9.5 % of patients in this cohort were experiencing a first ever episode of back and leg pain, although the majority were in the sciatica group. This is in line with evidence that back problems with or without leg pain are recurrent, with the majority of consulters reporting previous symptoms [23, 24]. This is also the case for patients with sciatica with an estimated 25 % reporting recurrence within 1 year of initial complaint [25]. However, in a secondary care cohort of patients with sciatica [26], 45.3 % reported first ever episode of sciatica. We think that this difference is most likely due to the question asked in the ATLAS cohort being both for back and/or leg pain, therefore we are not able to differentiate between the two complaints in terms of first episode.

Mean BMI in this cohort was 30, which is higher compared to the general population [27]. Analysis of longitudinal data will be more informative as to the relevance of this characteristic.

We used a self-reported neuropathic questionnaire to assess the presence of possible neuropathic pain. A higher proportion of patients in the sciatica group had scores indicative of neuropathic pain compared to the referred leg pain group. Sciatica is considered a type of peripheral neuropathic pain syndrome [28–30]. However, there is evidence indicating that sciatica (radiculopathy) may not necessarily be due to nerve damage in all cases [31, 32] and our findings that just under half of patients clinically diagnosed with sciatica had low scores on the neuropathic scales support this.

Disability mean level, as measured by the RMDQ, was higher (12.7) compared to other primary care consulting research cohorts with back pain with or without leg pain ((8.8) [1], (9.7) [33], (9.0) [34]), but lower compared with disc related sciatica patients enrolled in clinical trials where surgery is a treatment option (16.4) [35], as surgical candidates are expected to be at the worse end of symptoms spectrum. However, in contrast to other observational studies where sciatica patients reported higher disability levels compared to patients with referred leg pain [36, 37], in the ATLAS cohort both groups had similar levels. Pain related sleep disturbances were also commonly reported by this group at baseline, and similar to levels of sleep disturbance reported in a secondary care observational sciatica cohort (77 % vs 72 %) [26]. The self-reported impact of sciatic symptoms as assessed with the SBI, was 14.9 for the sciatica group, again similar to a secondary care sciatica cohort (14.2) [26]. The ATLAS cohort exhibited similar levels of anxiety (7.8) and depression (6.4) to other primary care cohorts visiting their GP (anxiety; 8.4, depression; 6.7) [1] or receiving physiotherapy for symptoms of back pain with or without leg pain (anxiety; 7.4, depression; 5.8) [33]. Within the ATLAS cohort, patients with sciatica and referred leg pain seemed similar in terms of anxiety and depression although the group diagnosed with referred leg pain had a higher proportion of anxiety and depression cases (see Table 4).

Overall, this study shows that primary care patients with back and leg pain have pain and impact approaching those seen in secondary care, and much worse than primary care back pain samples but with similar psychological status.

We used the STarT Back screening tool [17] to measure baseline risk of possible future poor outcome. Compared to other primary care research cohorts consulting with back pain with or without leg pain, only 13.9 % in the ATLAS cohort were at low risk of future disability, whereas the majority (56.8 %) of patients consulting their GPs with back pain with or without leg pain/sciatica are at low risk [1], this reduces to 26 % for patients receiving physiotherapy care for the same problems [33].

Approximately two thirds of the sciatica group and one third of the referred leg pain group had evidence of nerve root compression on MRI, with a disc prolapse being the most common finding in both cases. This is in line with well documented evidence that clinically diagnosed sciatic symptoms are not always supported by MRI findings and MRI evidence of nerve root compression may be present in the absence of any symptoms [38]. There is no ‘gold standard’ for the diagnosis of sciatica and it is, effectively, a clinical diagnosis where the clinician considers the pain is coming from a lumbar nerve root. As with all clinical diagnoses, the threshold for diagnosing sciatica may vary between clinicians [39]. An MRI showing nerve root compression should not be taken to confirm the diagnosis of sciatica but is simply one further piece of evidence that a clinician may take into account. However, MRI findings of equivocal or absent nerve root compression, whilst not excluding symptoms arising from the nerve root may change management options.

As this study aimed to identify everyone seeking care for symptoms of back and leg pain the findings can be used to estimate the general population prevalence of clinically assessed sciatica. Around 6 % of the UK general population visit their GP with back pain each year [40], and 61 % of these report having leg pain [1], representing 3.7 % of the general population. Combining this with the prevalence of sciatica of 74.2 % in the current study indicates that around 2.7 % of the general population have a clinical diagnosis of sciatica, each year. Although this estimate cannot account for selection bias, it is comparable to estimates from a systematic review on the prevalence of sciatica and supports the view that the prevalence of clinically assessed sciatica is lower (between 1.6 % up to 5 %) than estimates based on self-report (up to 43 %) [41].

### Strengths and limitations

The present study is the first to describe primary care patients seeking care for symptoms of low back and leg pain including sciatica without imposing selection criteria such as duration and severity, or strict diagnostic criteria/characteristics for defining the sciatica cases. Patients were recruited directly from general practice with computerised systems which maximises patient identification. This recruitment method, combined with the comparability of sciatica prevalence estimates (above), means that this cohort is likely to be representative of primary care patients seeking care for low back and leg pain.

Although attempts were made to maximise recruitment by using computerised identification, there was still the potential for patients to be missed, for example if the GP decided to override the system. This could lead to selection bias if invited patients differed from those who were not invited, although we do not have the data to investigate this.

Similarly, a number of invited patients did not attend or were not interested in participation. Age and gender characteristics for these non-participants are overall similar to those of the study’s participants. However, we do not have data on any of the other variables and therefore participation bias is a possibility if those who participated differed from non-participants on important characteristics (on which we have no data from non-participants).

Another potential limitation in describing the two groups (sciatica/referred leg pain) in this cohort is potential clinical diagnostic error. Diagnostic error and uncertainty is always a factor for a condition that can present with a wide spectrum of mild to severe symptoms. Diagnosis of sciatica is a probability of having the condition, based on clinical assessment findings. We captured diagnostic uncertainty by recording level of diagnostic confidence and over two thirds of the study patients were diagnosed with confidence of 80 % and above, which we consider as an acceptable level.

## Conclusions

In summary, in this unselected primary care cohort of patients seeking care for back and leg pain, disability levels are higher as compared with cohorts including mixed populations of LBP patients with and without pain in the leg(s) and similar for both sciatica and referred leg pain presentations. Nearly three quarters of the participants were clinically diagnosed as having sciatica. Approximately half of this cohort was likely to have pain of neuropathic nature as measured with self-reported scales. In contrast to non-specific LBP, minimal treatment was applicable to only a very small number of patients in this cohort. MRI findings of nerve root compression were present in just over half of the participants. There were differences between the sciatica and referred leg pain groups in terms of leg pain levels, neuropathic pain, bothersomeness due to the sciatic symptoms and MRI findings. Follow-up of this cohort will investigate the prognostic value of their baseline characteristics and explore the clinical relevance of the differences between those with sciatica and referred leg pain for the course of the low back and leg pain episode.

## Appendix

### Exclusion criteria

Less than 18 years of age

Red flags for serious pathology

Serious co-morbidity preventing patient from being able to attend the research clinic and undergo clinical assessment

Patients with serious mental problems who the GP considers to be vulnerable

Previous lumbar spine surgery

Pregnancy

Currently receiving physiotherapy or osteopathy or chiropractic or under care of secondary care consultant for the same problem

Not able to read and speak English

### Exclusion reasons

*Pre-clinic ineligibles;* n=38:

*Pre-clinic ineligibles;* n=38:

Already receiving treatment; n= 35

No symptoms; n=2

Previous spinal surgery; n=1

*Clinic ineligibles;* n=293:

Already receiving treatment; n= 27

No symptoms in leg; n=136

Previous spinal surgery; n=17

Not able to communicate in English; n=28

<18 years old; n=1

Patient too stressed to answer questions; n=1

Patient away during study; n=2

Leg pain not related to lumbar spine; n=21 (specific reasons given in 15 cases: muscular, knee pain, sacroiliac joint pain, peripheral neuropathy, vascular symptoms, widespread pain syndrome, meralgia parasthetica)

Hip problem; n=22

Suspected inflammatory arthropathy; n=4

Suspected sinister pathology; n=21

Patient unable to undergo examination; n=2

Severe mental problems; n=1

No reason stated; n=10

## Footnotes

1 The physiotherapists were qualified on average 19.5 years (range 7–41 years) and had an average of 15 years’ experience (range 6–27 years) in predominately treating musculoskeletal conditions.

2 In this paper, we report on three psychological constructs from the IPQ-R, timeline-acute/chronic; personal control, identity. This is based on existing literature indicating the distinctiveness of these three factors and their predictive relation to outcome (Foster et al. Pain (2010) 148:398–406).
